# Correction: Wali et al. Epigenetic Alterations in Hepatocellular Carcinoma: Mechanisms, Biomarkers, and Therapeutic Implications. *Pharmaceuticals* 2025, *18*, 1281

**DOI:** 10.3390/ph19070983

**Published:** 2026-06-25

**Authors:** Adil Farooq Wali, Abid Reza Ansari, Prince Ahad Mir, Mohamed El-Tanani, Rasha Babiker, Md Sadique Hussain, Jasreen Uppal, Asma Ishrat Zargar, Reyaz Hassan Mir

**Affiliations:** 1 RAK College of Pharmacy, RAK Medical and Health Sciences University, Ras Al Khaimah P.O. Box 11172, United Arab Emirates; 2 Laboratory of Epigenetics and Diseases, Department of Pharmacology and Toxicology, National Institute of Pharmaceutical Education and Research, S.A.S. Nagar (Mohali) 160062, Punjab, India; 3 Department of Pharmacognsoy and Phytochemistry, Khalsa College of Pharmacy, G.T. Road, Amritsar 143002, Punjab, India; 4 Department of Physiology, RAK College of Medical Sciences, Ras Al Khaimah Medical and Health Sciences University, Ras Al Khaimah P.O. Box 11172, United Arab Emirates; 5 Uttaranchal Institute of Pharmaceutical Sciences, Uttaranchal University, Dehradun 248007, Uttarakhand, India; 6 Pharmacology Division, Department of Pharmaceutical Sciences, University of Kashmir, Hazratbal, Srinagar 190006, Jammu and Kashmir, India; 7 Pharmaceutical Chemistry Division, Department of Pharmaceutical Sciences, University of Kashmir, Hazratbal, Srinagar 190006, Jammu and Kashmir, India

## References Correction

In the published manuscript [1], references 6, 16, 27, 156 and 165 have been replaced with the following references:

6.     Nair, M.; Sandhu, S.S.; Sharma, A.K. Cancer molecular markers: A guide to cancer detection and management. In *Seminars in Cancer Biology*; Academic Press: New York, NY, USA, 2018; Volume 52, pp. 39–55.
16.   Jindal, A.; Thadi, A.; Shailubhai, K. Hepatocellular carcinoma: Etiology and current and future drugs. *J. Clin. Exp. Hepatol.* **2019**, *9*, 221–232.
27.   Leclerc, J.; Flament, C.; Lovecchio, T.; Delattre, L.; Ait Yahya, E.; Baert-Desurmont, S.; Burnichon, N.; Bronner, M.; Cabaret, O.; Lejeune, S.; et al. Diversity of genetic events associated with MLH1 promoter methylation in Lynch syndrome families with heritable constitutional epimutation. *Genet. Med.* **2018**, *20*, 1589–1599.
156. Braghini, M.R.; Lo Re, O.; Romito, I.; Fernandez-Barrena, M.G.; Barbaro, B.; Pomella, S.; Rota, R.; Vinciguerra, M.; Avila, M.A.; Alisi, A. Epigenetic remodelling in human hepatocellular carcinoma. *J. Exp. Clin. Cancer Res.* **2022**, *41*, 107.
165. Hashemi, M.; Daneii, P.; Zandieh, M.A.; Raesi, R.; Zahmatkesh, N.; Bayat, M.; Abuelrub, A.; Koohpar, Z.K.; Aref, A.R.; Zarrabi, A.; et al. Non-coding RNA-Mediated N6-Methyladenosine (m6A) deposition: A pivotal regulator of cancer, impacting key signaling pathways in carcinogenesis and therapy response. *Non-Coding RNA Res.* **2024**, *9*, 84–104.

The authors state that the scientific conclusions are unaffected. This correction was approved by the Academic Editor. The original publication has also been updated.
